# The Predictive Value of Children's Understanding of Indeterminacy and Confounding for Later Mastery of the Control-of-Variables Strategy

**DOI:** 10.3389/fpsyg.2020.531565

**Published:** 2020-12-01

**Authors:** Sonja Peteranderl, Peter A. Edelsbrunner

**Affiliations:** ETH Zurich, Zurich, Switzerland

**Keywords:** control-of-variables-strategy, cognitive development, indeterminacy, confounding, longitudinal study, additive mixed models

## Abstract

Prior research has identified age 9–11 as a critical period for the development of the control-of-variables strategy (CVS). We examine the stability of interindividual differences in children's CVS skills with regard to their precursor skills during this critical developmental period. To this end, we relate two precursor skills of CVS at age 9 to four skills constituting fully developed CVS more than 2 years later, controlling for children's more general cognitive development. Note that *N* = 170 second- to fourth-graders worked on multiple choice-assessments of their understanding of indeterminacy of evidence and of confounding. We find relations between these two precursor skills and children's CVS skills 2 years later at age 11 in *planning, identifying*, and *interpreting* controlled experiments, and in recognizing the inconclusiveness of confounded comparisons (*understanding*). In accordance with the perspective that both indeterminacy and confounding constitute critical, related yet distinct elements of CVS, both precursor skills contribute to the prediction of later CVS. Together, the two precursor skills can explain 39% of students' later CVS mastery. Overall, the understanding of indeterminacy is a stronger predictor of fully developed CVS than that of confounding. The understanding of confounding, however, is a better predictor of the more difficult CVS sub-skills of understanding the inconclusiveness of confounded comparisons, and of planning a correctly controlled experiment. Importantly, both precursor skills maintain interactive predictive strength when controlling for children's general cognitive abilities and reading comprehension, showing that the developmental dynamics of CVS and its precursor skills cannot be fully ascribed to general cognitive development. We discuss implications of these findings for theories about the development of CVS and broader scientific reasoning.

## 1. Introduction

Scientific reasoning, which is typically described as a cyclic process of intentional knowledge-seeking through an empirical research process, encompasses skills such as generating and testing hypotheses, conducting controlled experiments, and the data-based evaluation of these experiments (Klahr, 2000; Kuhn et al., 2000; Wilhelm and Beishuizen, 2003; Zimmerman, 2007). Regarding experimentation, one crucial component is the control-of-variables strategy (CVS; Chen and Klahr, 1999). The CVS describes the technique to hold the levels of all variables constant except for the variable being investigated (Tschirgi, 1980; Chen and Klahr, 1999; Klahr, 2000; Dewey, 2002).

Although early developmental research indicated that children cannot develop understanding of CVS before early adolescence (Siegler et al., 1973; Tschirgi, 1980), later research indicated that precursor skills emerge already during childhood (Sodian et al., 1991; Bullock et al., 2009; Piekny and Maehler, 2013; Koerber and Osterhaus, 2019). We define precursor skills as the first emerging skills that build the foundation of more advanced and fully developed CVS. In this study, we examine whether and to what extent such precursor skills, in the present case children's understanding of indeterminacy of evidence and of confounding, can predict their mastery of more fully developed CVS skills 2 years later, and whether they have predictive value beyond children's general cognitive development.

### 1.1. Crucial Development Before Adolescence

Traditionally, educational and developmental researchers considered the experimentation skills of elementary school children to be deficient (Inhelder and Piaget, 1958; Klahr et al., 1993). The development of these skills was said to not emerge before adolescence (Inhelder and Piaget, 1958; Klahr et al., 1993). By contrast, recent research has revealed increasing evidence for a crucial period of development of the understanding of the CVS before adolenscence (e.g., Sodian et al., 1991; Bullock and Ziegler, 1999; Zimmerman, 2007; Bullock et al., 2009). Sodian et al. (1991) showed that already elementary school children could differentiate between controlled and confounded experiments. Bullock and Ziegler (1999) and Bullock et al. (2009) showed that children by the age of 8 years prefer controlled experiments over confounded experiments, but a spontaneous application of CVS has not been found (see also Zimmerman, 2007).

Specifically, Bullock and Ziegler (1999) delivered consistent findings on a task requiring the production and recognition of adequate tests for hypotheses and the relations between variables in the LOGIC study (Munich Longitudinal Study of the Genesis of Individual Competencies) with third to sixth graders. The children were asked to select from different comparisons that were either confounded or controlled to test a given hypothesis. By the age of ~8 years old, children preferred the conclusive comparisons over the confounded comparisons. In addition, more than 50% of the fourth graders, ~80% of the fifth graders and almost all of the sixth graders justified their choices by referring to the control of variables. Although children focus primarily on reasonable hypotheses (Klahr et al., 1993), they often recognize good comparisons if they are coherent with their initial beliefs (Sodian et al., 1991; Gopnik and Schulz, 2004; Croker and Buchanan, 2011), or they generate hypotheses to fulfill their prior theories (Kuhn et al., 1995; Schauble, 1996; Croker and Buchanan, 2011). These findings indicate some understanding of the CVS already in childhood, with increased development between 8 and 12 years of age.

This does however not imply that all individuals develop perfect CVS understanding before adolescence. Various studies (e.g., Kuhn, 2007) have shown that in some individuals, understanding of the CVS does not develop up to adulthood. Bullock et al. (2009) found that adults struggled with a metaconceptual understanding of alternative theories within experimental design (for review, see also Zimmerman and Croker, 2013; Zimmerman and Klahr, 2018).

Based on these results, we describe age 9–11 as a crucial developmental period. By crucial development period, we mean that increased development takes place during this period, and that there are shifts in the kinds of tasks that children are able to master before and after this period. The described evidence indicates that even some adults do not fully grasp the CVS. Hence, we presume that for some individuals who do not develop CVS during this period, further development might also be limited in the years after.

### 1.2. Sources of Development of CVS and Broader Experimentation Skills

There are at least three different sources that can contribute to children's development of CVS and broader experimentation skills. Even though not all children develop CVS on their own (Zimmerman, 2007), its development can be supported by implicit and explicit educational interventions (Chen and Klahr, 1999; Schalk et al., 2019). Schwichow et al. (2016b) summarized the findings from 72 CVS intervention studies and found a mean overall effect size of *g* = 0.61, with some studies indicating that even 6-year-olds can benefit from training.

Besides direct training, a more general factor that contributes to children's development of CVS is their general cognitive development. CVS and related experimentation skills do not develop fully independently of other cognitive abilities (for an overview, see Edelsbrunner et al., in Press). For example, CVS and related skills have been found to be associated with general reasoning skills (Mayer et al., 2014; Wagensveld et al., 2015), and with verbal skills and reading comprehension (Siler et al., 2010; Mayer et al., 2014; van der Graaf et al., 2016; Osterhaus et al., 2017).

Finally, various skills related to CVS and broader scientific reasoning might represent sources of development for each other through mutual developmental relations. CVS and further aspects of scientific reasoning themselves are usually intercorrelated (e.g., Mayer et al., 2014). Such interrelations relate to the question of whether scientific reasoning should be described as a unidimensional construct, or as a construct that incorporates multiple dimensions that operate and develop in parallel (Zimmerman, 2007). Koerber et al. (2015) proposed a conceptual model based on the idea that the core of scientific reasoning is the ability to differentiate and coordinate theories and evidence (Kuhn, 2010). Based on this idea, they predicted a unidimensional psychometric structure of scientific reasoning, describing evidence for this notion in Mayer et al. (2014) and Koerber et al. (2015), and Koerber and Osterhaus (2019) based on finding adequate itemfit in Rasch analyses. Edelsbrunner and Dablander (2018), however, based on data simulations and psychometric considerations, argued that itemfit provides limited information for distinguishing a common core from further sources of intercorrelations between different skills related to scientific reasoning. Testing different assumptions about the cognitive abilities underlying a set of intercorrelated tasks is generally very difficult based on cross-sectional data (van Bork et al., 2019; VanderWeele and Batty, 2020). More fruitful information regarding the question of whether it is useful to model skills related to scientific reasoning unidimensionally or multidimensionally might be gathered from looking into the developmental interplay of multiple skills over time. For example, if multiple skills related to scientific reasoning show additive or interactive developmental and predictive patterns regarding later skills, this would imply that the former skills should be conceptualized and modeled separately. If, however, multiple skills related to scientific reasoning exhibit interchangeable developmental patterns or predictive value for later skills, treating these as a unitary construct would not imply loss of information.

### 1.3. Different Sub-skills of CVS

Different types of tasks have been used to assess CVS, with tasks that assess similar skills sometimes receiving different labels across studies. In the present study, we follow a distinction of CVS sub-skills that has been developed by Schwichow et al. (2016a) based on a definition of CVS used in a seminal study by Chen and Klahr (1999).

According to the distinction by Schwichow et al. (2016a), CVS incorporates four sub-skills: *interpreting* controlled experiments, *identifying* controlled experiments, *understanding* the indeterminacy of confounded comparisons, and *planning* controlled experiments. More specifically, *interpreting* describes the ability to interpret a controlled experiment based on the outcome. *Identifying* describes the ability to select a suitable comparison according to a specific hypothesis; *understanding* declares knowledge about the indeterminacy of confounded experiments; in other words, the knowledge that no valid conclusion can be drawn from a confounded experiment. *Planning* describes the capability to build a comparison according to a given initial hypothesis based on provided variables. A comparison between the labels that were given to tasks that involve similar skills helps elucidate the differences between the four sub-skills, and why these distinctions in our perspective are informative.

In a recent study, Koerber and Osterhaus (2019) labeled tasks in which children had to interpret controlled or confounded experiments *data interpretation*. In the distinction by Schwichow et al. (2016a) and our study, these tasks would fall under two different categories. When children have to draw the correct conclusion from a controlled experiment, we call the relevant CVS sub-skill *interpretation*. In the case in which children have to interpret a confounded comparison, however, they have to understand that no conclusive conclusion is possible because of the confounding. We call the relevant CVS sub-skill *understanding*. Schwichow et al. (2016a) found that the latter appears to be much more difficult for students. In a more recent study, Schwichow et al. (2020) found that understanding is the most difficult sub-skill and in analyses of subgroups of students, they found that it might premise mastering the other more procedural sub-skills. Given the differential difficulty and information that these two sub-skills seem to provide, we also distinguish between the controlled and confounded cases and the involved sub-skills.

The remaining two sub-skills, *identifying* and *planning*, can be distinguished by comparing them to tasks in the longitudinal study by Bullock et al. (2009). In tasks that Bullock et al. (2009) labeled choice-tasks, children had to select the correct, controlled comparison among different proposals for setting up an experiment. In the present study, we refer to this kind of task as assessing the precursor skill of confounding we will further explain this in the next section, and in our conceptualization of fully developed CVS it receives the label *identifying*. Hence we see this as a development of the precursor to a fully developed CVS sub-skill. In tasks that Bullock et al. (2009) labeled production-tasks, children had to actively set up experimental comparisons on their own. In the present study, such tasks receive the label *planning*. Bullock et al. (2009) and further studies (see Zimmerman, 2007) found that production tasks were more difficult and children's underlying skills developed later than those on choice-tasks. We therefore also distinguish between these two different tasks and the underlying sub-skills.

### 1.4. Precursor Skills of CVS

With regard to the kinds of tasks that children can solve, based on prior findings we distinguish between precursor skills of CVS that many children develop in earlier childhood, and fully developed CVS that typically does not develop before this period. Two precursor skills of more fully developed CVS are the understanding of indeterminacy and that of confounding.

Indeterminacy refers to the understanding of whether available evidence is sufficient to warrant a specific conclusion (Klahr and Chen, 2003). For example, when a toy consists of plugged round parts and the question is which of two boxes the parts were taken from, but both boxes contain round parts, then the evidence is indeterminate (Piéraut-Le Bonniec, 1980). In experimental design, the indeterminacy principle pertains to the focal variable about which the causal status is in question. In order to produce determinate evidence, the focal variable has to be varied such that it can yield determinate evidence. An example for indeterminate experimental evidence is when an object that smells strongly to humans is hidden to find out whether a German shorthaired pointer-dog can smell better than humans: Even humans could have smelled the object; therefore, if the dog finds it, this does not denote determinate evidence, and no conclusion can be drawn. Indeterminacy makes the first puzzle piece to later mastery of CVS: Before the control of confounding variables is considered, the right focal variable has to be varied in a manner such that conclusions about the question at hand will be possible. Piekny and Maehler (2013) investigated the understanding of indeterminacy by asking children to design an experiment, employing a task by Sodian et al. (1991). They asked 4- to 12-year-old children to choose between two mice houses one with a big opening and one with a small opening in order to feed both (the small and the large) mouses (problem 1) and find out whether the mouse that went inside to eat was either big or small (problem 2). Their results showed that more than 50% of the 5-year old children could solve correctly problem 1 but failed in solving problem 2.

A second precursor skill is the understanding of confounding. This is the second step toward fully developed CVS: In addition to correctly varying the focal variable, confounding variables have to be controlled. As precursor skill, we refer here to recognition of situations in which confounding variables are correctly controlled, or not. Multiple studies have shown that such recognition can be triggered in many children in simple tasks, whereas active or spontaneous application of variable control is comparably rarely found in children before the outlined crucial developmental period before adolescence that we have explained in the introduction (Kuhn, 1989; Bullock and Ziegler, 1999; Bullock et al., 2009; van der Graaf et al., 2015; Koerber and Osterhaus, 2019).

Although the understanding of indeterminacy and confounding represent two early facets of CVS, mastery of CVS encompasses all four sub-skills. These require more active production and deep conceptual understanding of the role of CVS. In the present study, we examine the predictive value of these two precursor skills for mastery of CVS encompassing the four sub-skills 2 years later.

### 1.5. The Present Study

The present study examined to which degree children's understanding of *indeterminacy* and of *confounding* can predict their mastery of the CVS 2 years later, after most of the crucial developmental period for CVS. We also examined whether the developmental patterns taking place during this period can be discerned from children's more general cognitive development. Specifically, we asked (1) to what extent the understanding of indeterminacy and confounding can predict later mastery of CVS, (2) whether the predictive strength of the precursor skills remains robust when taking into account children's general cognitive abilities and reading comprehension as covariates, (3) whether and how the precursor skills' predictive strength differs between the four CVS sub-skills of *planning, identifying*, and *interpreting* controlled experiments, and recognizing the inconclusiveness of confounded experiments (*understanding*), and again checking (4) whether the predictive relations hold when taking into account the covariates.

To this end, we assessed a sample of primary school children from Switzerland twice. The sample was part of the Swiss MINT Study, a large-scale longitudinal study on the effects of early Physics education conducted at ETH Zurich (for more details see https://educ.ethz.ch/lernzentren/mint-lernzentrum/Schweizer_MINT_Studie.html). The tasks applied in the present study to assess CVS and its sub-skills stem from Peteranderl (2019), who developed and evaluated a new inventory for measuring experimentation skills with a focus on fifth and sixth graders. This inventory covers the assessment of all four sub-skills of CVS, as summarized by Schwichow et al. (2016a), among the assessment of other skills of scientific reasoning, such as scientific argumentation or failure in experimentation because of incorrect preconceptions about experimentation, as summarized by Schauble et al. (1991) and Siler and Klahr (2012).

In the present study, the children were in second to fourth grade and on average 9 years old at the first assessment, meaning that this assessment took place in the beginning of the crucial developmental period 9–11. The second assessment took place 2 years later, when children were in fifth to sixth grade, about 11 years old on average, such that this assessment took place toward the end of the crucial developmental period.

At the first assessment, we assess children's understanding of indeterminacy and confounding, and at the second assessment the four CVS sub-skills, as well as general reasoning and reading comprehension. With this study design, we aimed at estimating the stability between the precursor skills and later CVS. Do children remain relatively stable from the precursor skills to later mastery of CVS, or do new substantial individual differences emerge during the crucial developmental period? In addition, we examine general cognitive abilities and reading comprehension, in order to test whether the predictive value of the two precursor skills represents specific dynamics that are distinct from children's more general cognitive development.

## 2. Materials and Methods

### 2.1. Sample

The sample stemmed from two separate measurement points with a total sample size of 170 primary school children (90 female, 80 male) from 12 school classes in the German-speaking part of Switzerland. The children were in second (*n* = 48), third (*n* = 54), or fourth (*n* = 68) grade at the first assessment (*M*_*age*_ = 9.07, *SD* = 0.95), and in fifth (*n* = 133) or sixth (*n* = 37) grade at the second data assessment (*M*_*age*_ = 11.18, *SD* = 0.46). There was a median time gap (we report medians for skewed data) of 23 months between the two data assessments, with a range of 10–34 months.

### 2.2. Assessment of Precursor Skills

The assessments took place within the framework of the Swiss MINT Study, a longitudinal study investigating the impact of early science education on children's later academic and cognitive development (Edelsbrunner et al., 2015, 2018; Schalk et al., 2019).

At the first assessment (when children were in the second, third, or fourth grade; for an example, see 1), the children were assessed with the precursor skills assessment. This tool was a questionnaire encompassing 10 multiple-choice items (internal consistency reliability estimate: McDonald's omega = 0.81; for an explanation of omega; see Dunn et al., 2014) based on typical scenarios from the CVS literature (e.g., the Airplane- and Ramp-tasks; Bullock and Ziegler, 1999; Chen and Klahr, 1999) and similar scenarios.

The understanding of *indeterminacy* as one of two precursor skills was assessed by four items. Each of these items presented the children with a short story including the research question (e.g., “Which giraffe ate the carrot?”) and each of the outcomes under different manipulations of the focal variable (e.g., “the big giraffe with her long neck can reach the tips of all trees”). In a second step, the children were asked to select the correct variable in order to answer the initial research question by choosing the correct response within a multiple-choice response format. Children's mean score of correct answers on these items was used for analyses.

*Confounding* as the second precursor skill was assessed by six items. In these items, children were faced with a short story including different variables containing two levels each. In order to investigate a given research question referring to exactly one out of these variables (e.g., “what should Mr. Miller do to find our whether the form of the nose is important for how much fuel the airplane needs”), the children were then asked to select the correct response out of three possible multiple-choice responses. Children's mean score of correct answers on these items was used for analyses.

**Figure 1:**
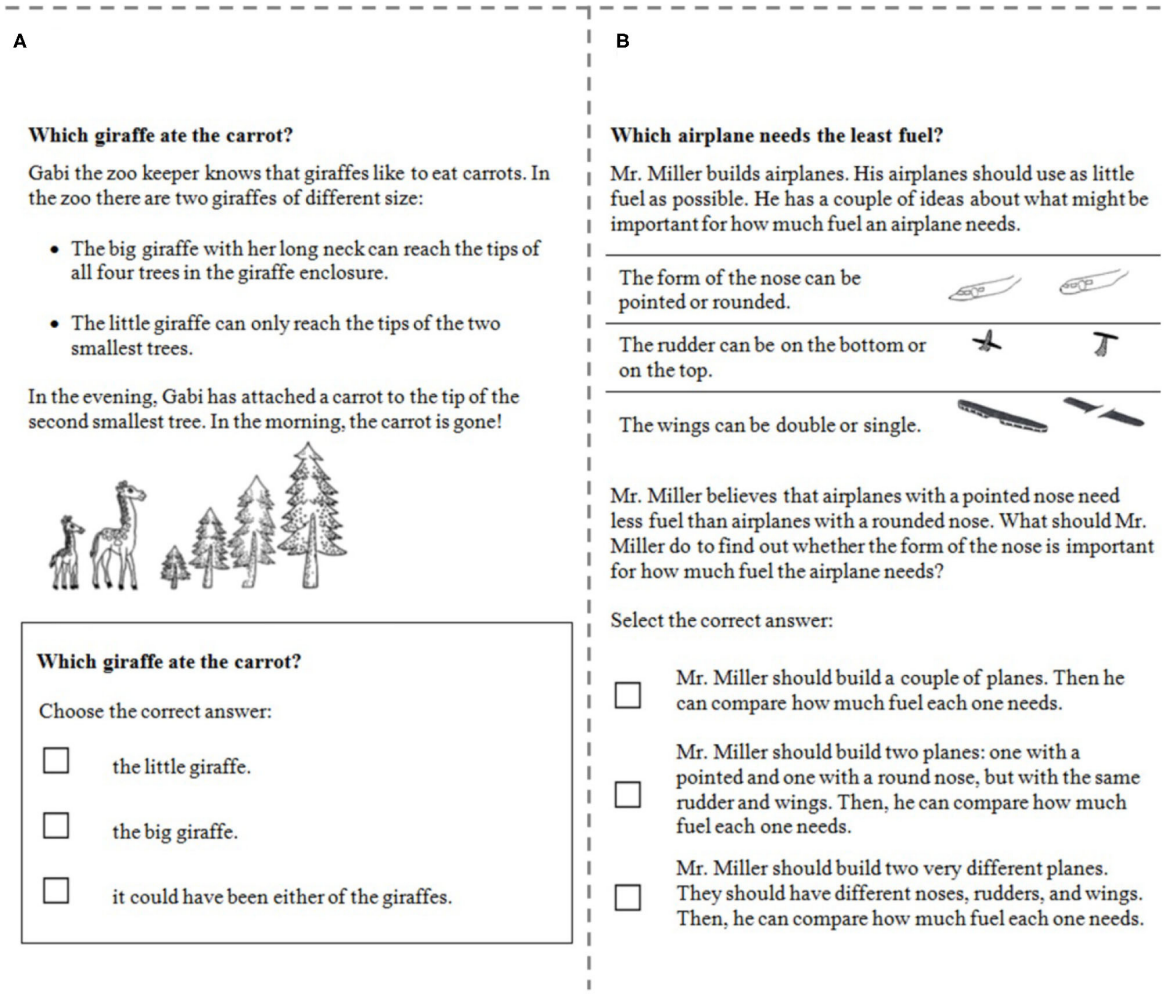
Example items from the assessment of the precursor skills of understanding indeterminacy **(A)** and confounding **(B)**.

Figure 1 shows an example item of each precursor skill, indeterminacy (Figure 1A) and confounding (Figure 1B). The items have been validated and used in prior research on the development of CVS and the impact of educational interventions (Edelsbrunner et al., 2015, 2018; Schalk et al., 2019).

### 2.3. Assessment of the Control-of-Variables Strategy

**Figure 2:**
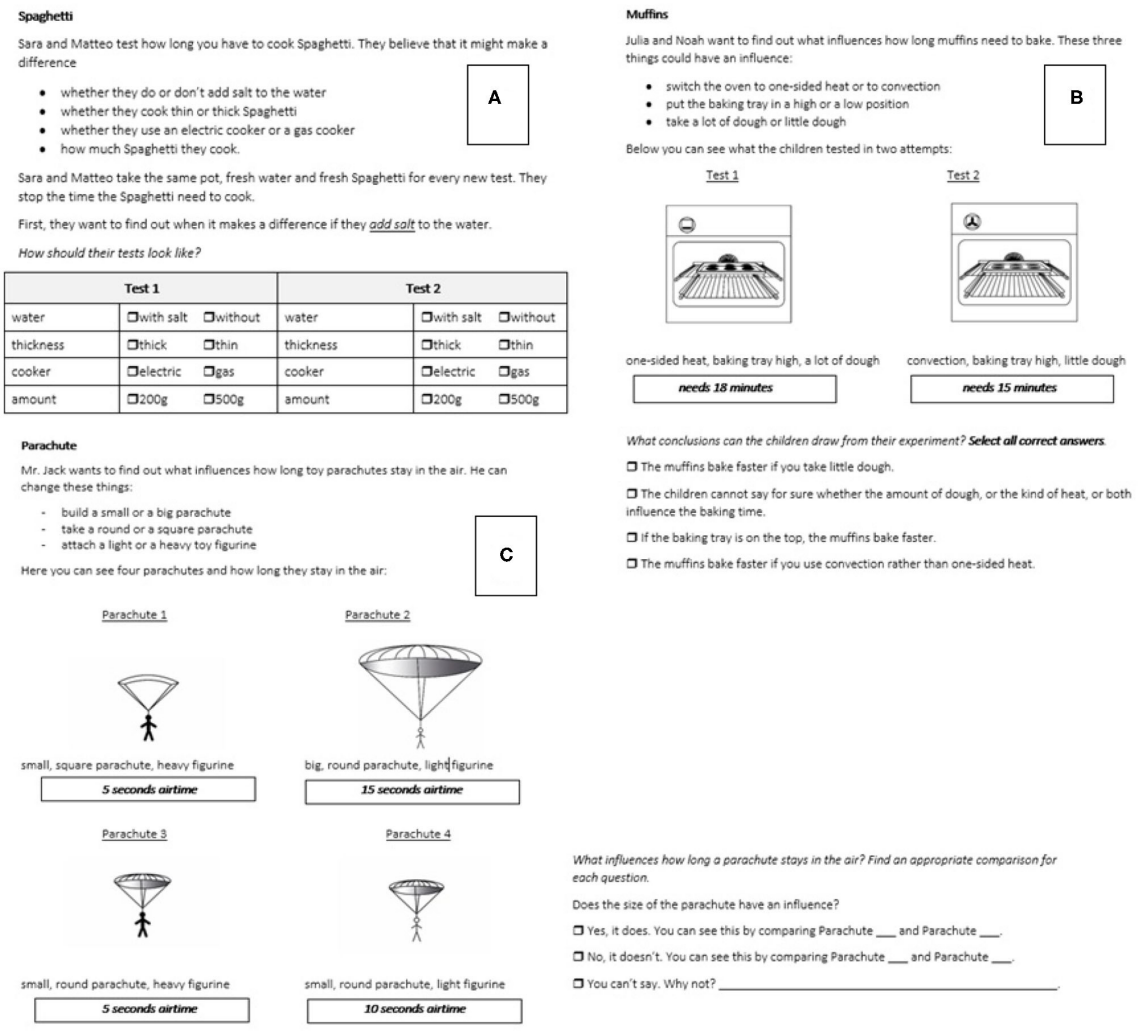
Example items for the assessment of each sub-skill of CVS. The items on top assess the sub-skills *planning* **(A)** and *understanding* **(B)**. The item at the bottom **(C)** assesses the sub-skills *interpreting* and *identifying*.

At the second assessment (when children were in the fifth or sixth grade), the children were assessed with the CVS assessment. This tool stemmed from prior research investigating the impact of a CVS intervention in Swiss school children (Peteranderl, 2019). The assessment was a questionnaire encompassing 15 items with similar contexts as in the first tool (internal consistency reliability estimate of the overall test: omega = 0.84, of the sub-skill *interpreting*: omega = 0.91, of *identifying*: omega = 0.91, *understanding*: omega = 0.93, and *planning*: omega = 0.85). In this tool, the focus was on assessing all four sub-skills of CVS that can be distinguished based on the definition of Schwichow et al. (2016a) separately within different types of items. In Figure 2, items for the assessment of all four sub-skills of CVS are presented.

The CVS assessment encompassed four types of items for the assessment of the four sub-skills. However, children's solutions on the different item types contributed to scores of multiple of the four sub-skills. We first describe the four item-types, and then how children's scores on the four sub-skills were composed based on these. In all items, the children are faced with a research question and a hypothesis describing three to four different variables with two levels each. In the first type of items, *planning* (Figure 2A), the children are then asked to select the correct levels of four given variables according to the initial hypothesis in order to plan a conclusive experiment. In the second item type, *understanding* items (Figure 2B), the children are faced with an initial hypothesis and a suitable, but confounded experiment two variables are varied (instead of one variable) out of three given variables. They are asked to detect the inconclusiveness of this experiment and to select the correct response option reflecting the indeterminacy of confounding (“The children cannot tell for sure...”). In the third and fourth types of items, the *interpretation* and *identifying* items (Figure 2C), the children are faced with a research question related to three variables of interest. They are presented with four different sketches of four attempts to set up an experiment for examining the research question. The children are asked to select a suitable comparison to test the hypothesis (each item presents three hypotheses about the impact of the three variables on the outcome) reflecting the sub-skill *identifying*. The children are also asked to interpret the results of the outcome of the experiment with regard to the respective hypothesis, reflecting the sub-skill *interpreting*.

An overall CVS score of the second assessment was calculated based on all the items assessing the four CVS sub-skills, and four additional items. In the additional items, the children were asked for short written justifications about their reasoning in interpreting experiments. Ratings of these justifications together with children's scores on the four CVS sub-skills yielded an overall CVS score with a maximum of 35 points.

The scores for the four sub-skills were generated based on children's responses on the four described item types. The interpretation items contributed to the assessment of three sub-skills: *Interpreting* was constructed by drawing valid inferences from unconfounded comparisons in 10 cases. *Identifying* was constructed by the correct choice of an unconfounded comparison in the interpretation items in 10 cases. *Understanding* was constructed based on the detection of the indeterminacy of a confounded experiment in all of the interpretation items (in total 2 cases) and in all of the understanding items (in total 3 cases). The sub-skill *planning* was constructed by selecting a controlled experiment with regard to the initial hypothesis in the planning items (4 cases). The CVS assessment has been validated psychometrically, in cognitive interviews, and by asking students for additional open answers in order to probe construct validity (Peteranderl, 2019).

### 2.4. Covariates: Cognitive Abilities and Reading Comprehension

At the second assessment, we additionally measured children's general cognitive abilities and reading comprehension with standardized instruments. For measuring cognitive abilities, we used the numerical and figural analogies scales of the Germany-wide established cognitive abilities test for primary school children [Kognitiver Fähigkeitstest (KFT); Heller and Perleth, 2000]. In the numerical scale, students are presented with 20 items, each containing 4–5 numbers in a row following a specific rule. The students must ascertain this rule by recognizing the correct number continuing the row out of five provided answers (numbers). For this scale, 9 min was scheduled. In the figural scale, students are presented with 25 items containing pairs of figures that are related according to a specific rule that the student must determine. Afterwards, students must choose one of five provided answers (figures) that fits with another figure according to this rule. For this scale, 8 min was scheduled. Internal consistency reliability estimates were omega = 0.88 for the numerical scale and omega = 0.92 for the figural scale. The overall sum score from both scales was used as a covariate representing reasoning ability in the main analyses.

For measuring students' reading comprehension, we used the latest version of a standardized test instrument for fifth graders [Lesegeschwindigkeits-und-verstndnistest (LGVT, 2. Auflage); Schneider et al., 2017]. In this test, students have to read a text containing 2,161 words as far as they can within 6 min to measure reading speed. The text contains gaps with missing words that the students have to fill in by choosing the correct word from a selection of three words, measuring reading comprehension and reading accuracy. The internal consistency reliability estimate in this sample was omega = 0.80.

## 3. Results

### 3.1. Descriptive Statistics

The descriptive statistics and intercorrelations of the main study variables are provided in Tables 1, 2. The main study variables comprise the two precursor variables *indeterminacy*, understanding of conclusive testing, and *confounding*, understanding the control of nonfocal variables, at the first assessment. The main variables at the second assessment comprise the four sub-skills of CVS: *interpreting, identifying, understanding*, and *planning*, as well as overall *CVS* mastery and the two covariates.

**Table 1:** Descriptive statistics of the main study variables.

	**Maximum score**	**Mean (in %)**	**SD**
**First assessment**			
Indeterminacy	1.00	0.54	0.30
Second grades (*n* = 48)		0.43	0.25
Third graders (*n* = 54)		0.60	0.31
Fourth graders (*n* = 68)		0.57	0.30
Confounding	1.00	0.37	0.27
Second grades (*n* = 48)		0.31	0.22
Third graders (*n* = 54)		0.45	0.31
Fourth graders (*n* = 68)		0.34	0.27
**Second assessment**			
CVS	35.00	13.58 (39%)	7.29
Identifying	10.00	4.33 (43%)	3.22
Interpreting	10.00	6.64 (66%)	2.81
Planning	4.00	0.98 (25%)	1.50
Understanding	5.00	0.54 (11%)	0.88
Cognitive abilities	45.00	30.44 (68%)	10.51
Reading comprehension	47.00	15.07 (32%)	10.06

*Scores for separate school grades presented for indeterminacy and confounding. Scores for indeterminacy and confounding were scaled as mean scores ranging from 0 to 1, such that their mean scores can be interpreted as percentages, whereas scores on other measures indicate sum scores, with percentages in brackets*.

**Table 2:** Intercorrelations of the main study variables.

	**Indet**	**Confo**	**CVS**	**Inter**	**Ident**	**Under**	**Plann**	**CogAb**
Indeterminacy								
Confounding	0.47							
CVS	0.55	0.42						
Interpreting	0.34	0.17	0.73					
Identifying	0.46	0.30	0.85	0.54				
Understanding	0.28	0.33	0.49	0.22	0.20			
Planning	0.46	0.42	0.68	0.25	0.47	0.33		
CogAb	0.35	0.25	0.46	0.36	0.38	0.15	0.34	
ReadComp	0.37	0.27	0.52	0.34	0.44	0.20	0.42	0.48

*Indet, indeterminacy; Confo, confounding; Inter, interpreting; Ident, identifying; Under, understanding; Plann, planning; CogAb, cognitive abilities; ReadComp, reading comprehension*.

**Figure 3:**
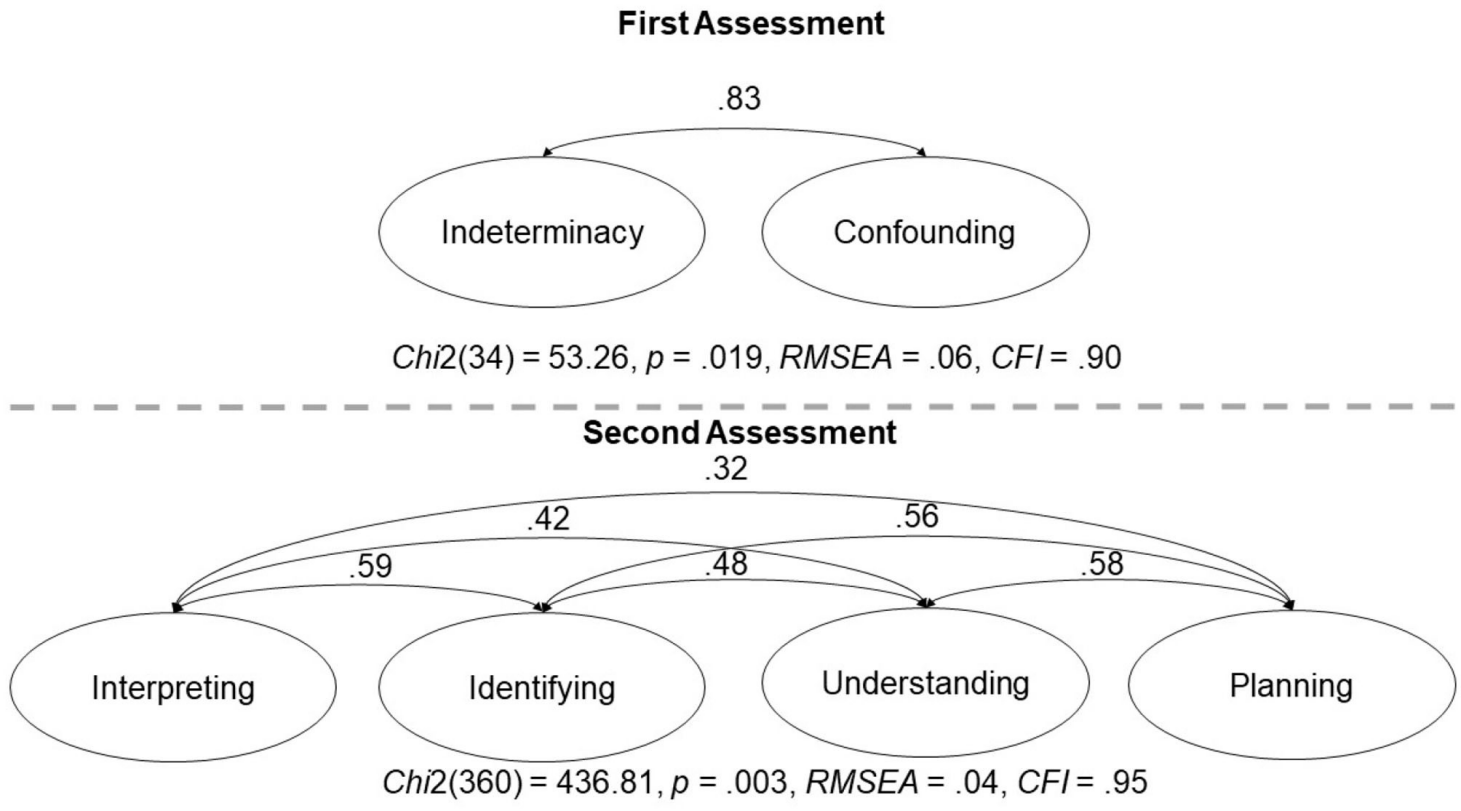
Results from confirmatory factor analyses for the skills at the first (upper part) and second assessments (lower part), respectively. Indicator variables (individual items) omitted for visual clarity. All intercorrelations were significant at *p* < 0.001. When fixing the intercorrelation between inderterminacy and confounding to 1, the CFI deteriorated slightly to 0.89, and fixing any of the intercorrelations between the four CVS sub-skills from the second assessment to 1 substantially worsened model fits.

In order to check that the distinctions between the two precursor skills at the first assessment, and between the four CVS sub-skills at the second assessment were psychometrically valid, we conducted two confirmatory factor analyses. In these analyses, we had the items load onto two (first assessment) or four (second assessment) latent variables representing the precursor skills and the four fully developed CVS sub-skills. The results of the analyses, depicted in Figure 3, indicated that the measurement models fit sufficiently well and that the two or four latent variables showed moderate to substantial intercorrelations, which however were below 1.

### 3.2. The Predictive Strength of Precursor Skills for Later CVS

**Figure 4:**
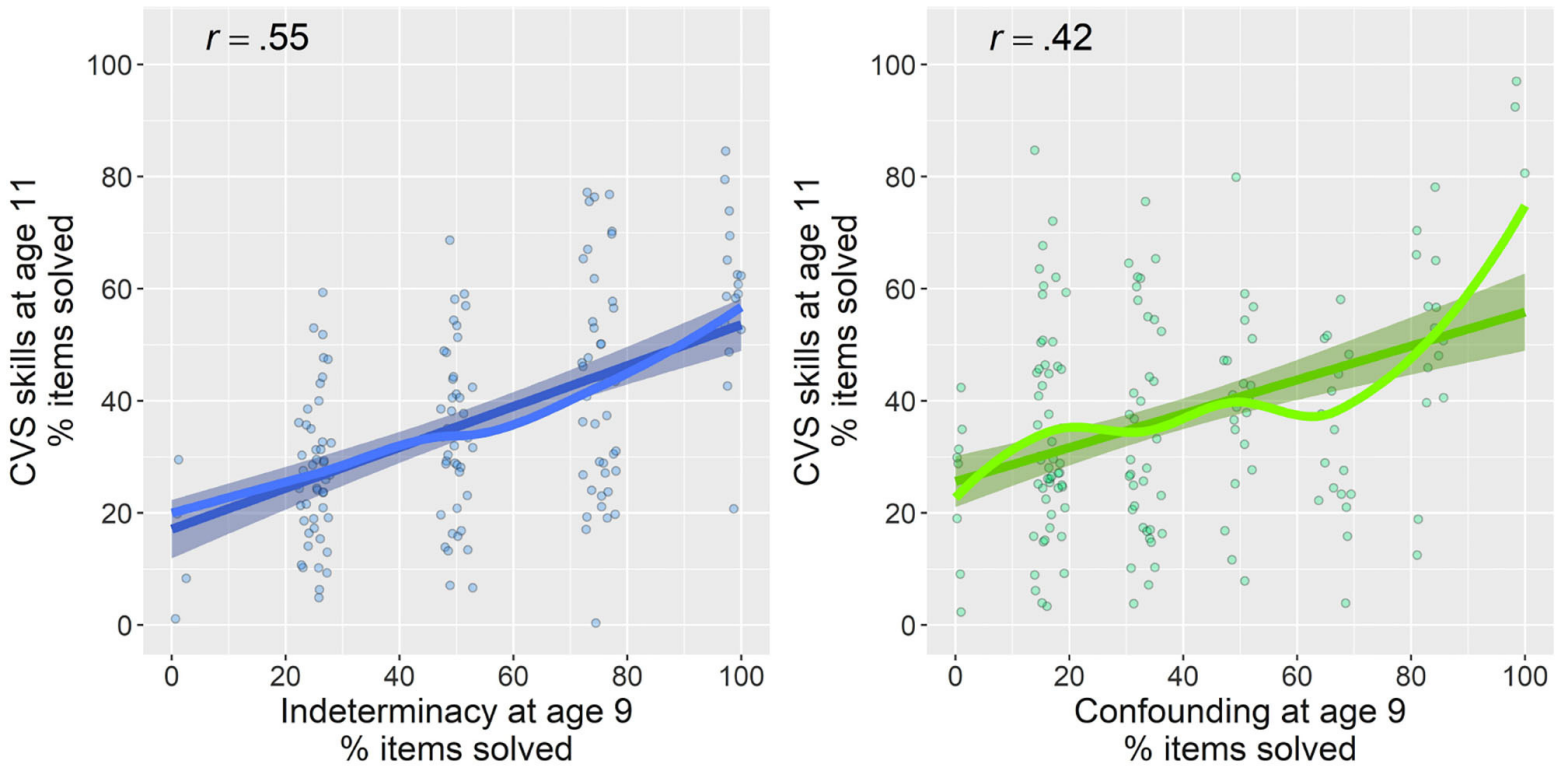
Relations of indeterminacy **(left)** and confounding **(right)** at average age 9 with later overall CVS skill at average age 11. Percentage of items solved are plotted for all measures. Lines with 95% confidence band indicate linear trend, and lines without confidence bands indicate nonlinear (smooth) associations. Points are jittered for a better readability of scatterplots. Pearson correlations between variables are displayed in upper-left of each plot.

In order to examine the predictive strength of the two precursor skills for children's overall sum score on later CVS, we first investigated scatter plots (Figure 4). These indicated partially nonlinear relations between the two precursor skills and CVS. We therefore estimated additive mixed models, a regression technique that allows multilevel modeling, and particularly the capturing of nonlinear relations, whereas avoiding adjusting predictive terms unduly toward data outliers (Groll and Tutz, 2011; Wood, 2017). We fitted four models, the fit and explained variance of which are summarized in Table 3. We first estimated models with indeterminacy and confounding, respectively, as individual predictors for later CVS (Table 3: Models 1 and 2). We did not yet take into account classroom dependencies, because we were first of all interested in the predictive power of the two precursor skills when not yet deducing classroom differences that might overlap with the variances from these estimates. The two models indicated that indeterminacy alone could explain 29.7% of children's later CVS mastery (*F* = 37.12, *p* < 0.001), whereas confounding alone could explain 25% (*F* = 11.83, *p* < 0.001; for relative model fits, see Table 3).

**Table 3:** Relative model fit and explained deviance for the four fitted general additive (mixed) models.

**Model no**.	**Model description**	**AIC**	**BIC**	**% deviance explained**
Model 1	Indeterminacy	1,460	1,472	29.7
Model 2	Confounding	1,474	1,495	24.9
Model 3	Indeterminacy × confounding	1,441	1,469	39.2
Model 4	Indeterminacy × confounding, covariates, teacher	1,393	1,505	61.6

*Covariates were reasoning ability and reading comprehension, including their interactions with indeterminacy and confounding. teacher indicates a random intercept across teachers/classrooms*.

**Figure 5:**
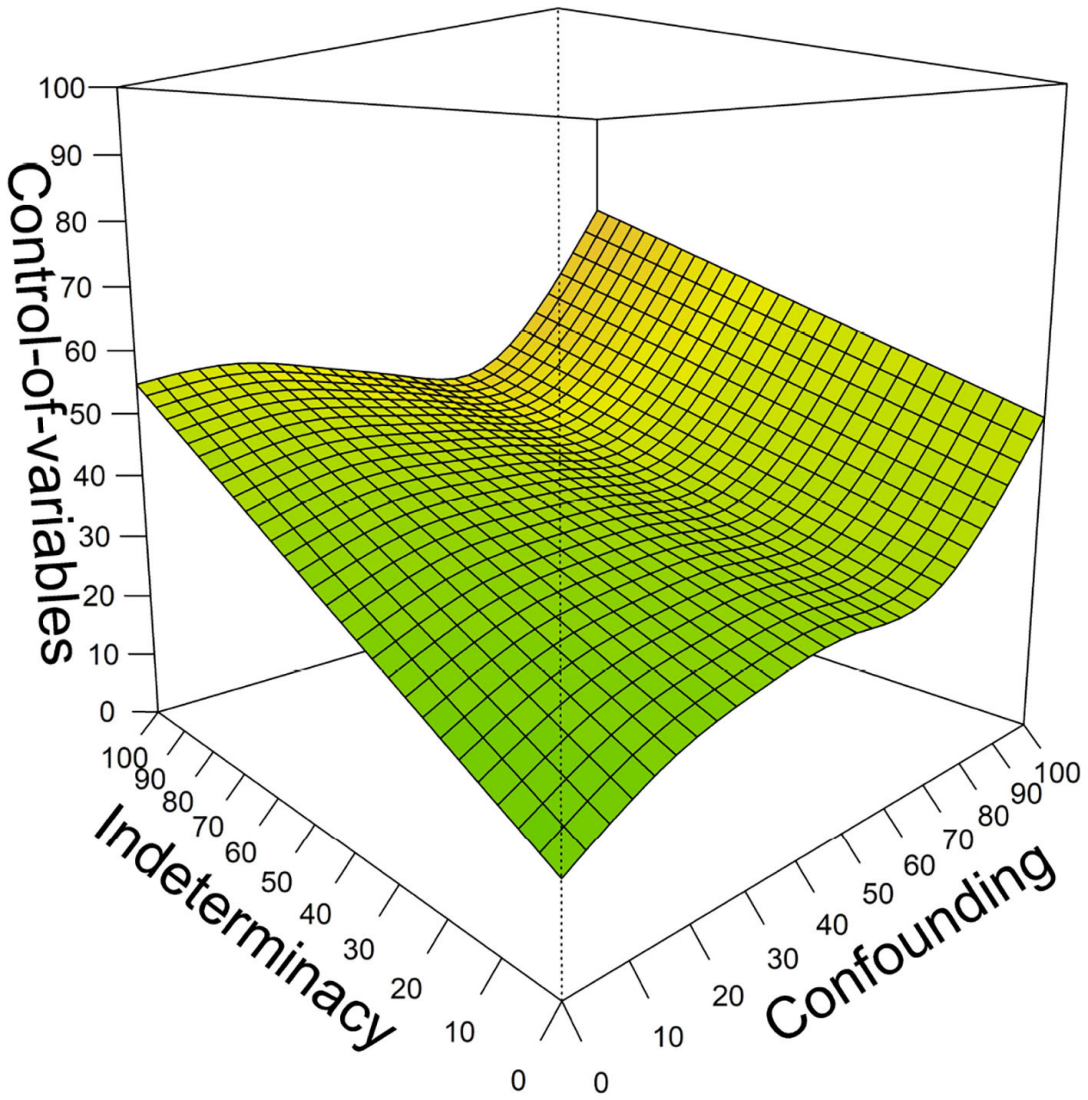
The prediction of children's mastery of CVS from their understanding of indeterminacy and confounding 2 years earlier. All axes indicate model estimates of percentage of solved items on the respective measure. Areas in darker green indicate lower predicted later CVS skill, areas in brighter green moderate, and in orange highest later CVS skill.

Combining both predictor variables and adding an interaction between the two variables (Table 3: Model 3) showed a small interaction (*F* = 0.244 *p* = 0.035) and main effects for both variables (indeterminacy: *F* = 36.85, *p* < 0.001, confounding: *F* = 4.72, *p* = 0.001), indicating that both variables add to the prediction of CVS beyond each other. In this combined model, both variables together managed to explain 39.2% of variation in later CVS mastery. We consider this model the most important one for our research question, because it shows the predictive power of the two precursor skills in combination. The relation of the two precursor skills with children's later CVS based on this model is depicted in Figure 5. A three-dimensional depiction is provided for visualizing the combined predictive value of both precursor skills. The figure can be read as follows: Children who are low on both predictor variables (dark area in lower front; both x- & y-axes close to 0) are estimated to solve about 20% of the later CVS items (z-axis). For children with low levels of indeterminacy (lower area of percentage solved on the axis labeled “Indeterminacy”), confounding has substantial positive predictive value: If children at least manage to solve many confounding-items, they are predicted to have relatively good estimated CVS-skill later on (about 50% solved items predicted; area high on confounding but low in indeterminacy in the right). The same holds vice versa: Children lower on the axis labeled “Confounding” but higher on the “Indeterminacy” axis have relatively good predicted CVS skill 2 years later, with about 55% solved items. The highest predicted later CVS skill, however, is reached by children who are high on both axes (area shaded in orange); for these children, estimates of later overall CVS skill are at about 80%. The interaction between the two predictor variables is for example visible for children who achieve moderate scores on confounding but low scores on indeterminacy; for these children, the estimated CVS skill 2 years later is higher than purely additive effects would indicate. Consequently, the surface within this area appears slightly elevated in comparison to the remaining surface patterns.

**Figure 6:**
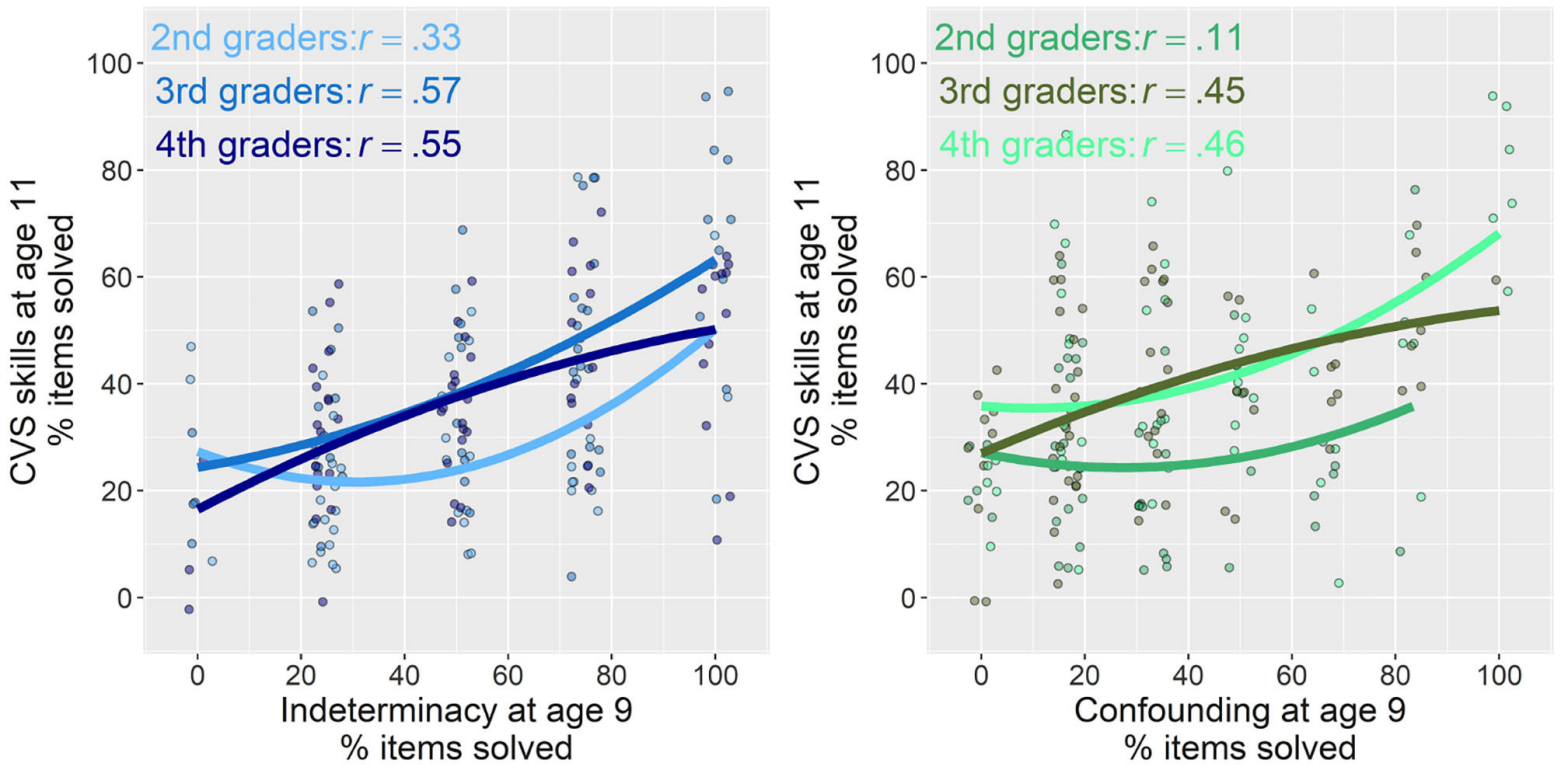
Relations of indeterminacy **(left)** and confounding **(right)** at average age 9 with later overall CVS skill at average age 11 separate for the grades in which the first assessment took place. Pearson correlations between variables are displayed in upper left of each plot, whereas the regression lines indicate quadratic fit for a good balance of information and readability.

We added visual and inferential robustness checks to examine whether the grade in which children underwent the first assessment and the lag between the first and second assessment influenced these results. As visible from Figure 6, relations between the two precursor skills with later CVS were less strong if the first assessment took place already in second rather than third or fourth grade. Relations did not seem to differ, however, between those for whom the first assessment took place in third or fourth grade. Adding an interaction between the predictor terms of indeterminacy and confounding and the time lag between the first and second assessment did not show any interactions, both *p* > 0.10.

Finally, we examined the robustness of the predictive effects regarding children's covariates, as well as taking into account the classroom structure. We added a random intercept across teachers in order to control the intercept for the multilevel structure, and main effects of reasoning ability and reading comprehension (including their interactions with main effects; Simonsohn, 2019) to the model (Table 3: Model 4). In this model, controlling for classroom dependencies and the two covariates, the effects of indeterminacy and confounding became smaller, however the interaction effect, in particular, indicated that both variables still had predictive value beyond the variance shared with the classroom differences and covariates (indeterminacy: *F* = 20.27, *p* < 0.001; confounding: *F* = 1.94, *p* = 0.100; interaction: *F* = 0.48, *p* = 0.014).

Overall, these analyses indicate that both indeterminacy and confounding are substantial predictors of later CVS mastery and together they explain about 40% in the variance of later CVS (Table 3: Model 3). In addition, throughout the models, indeterminacy appeared as an overall stronger predictor of later CVS than confounding; however, both variables had predictive value beyond each other that remained intact when taking into account classroom dependencies and children's general cognitive development.

### 3.3. Variation in Predictive Strength Across Sub-skills

**Figure 7:**
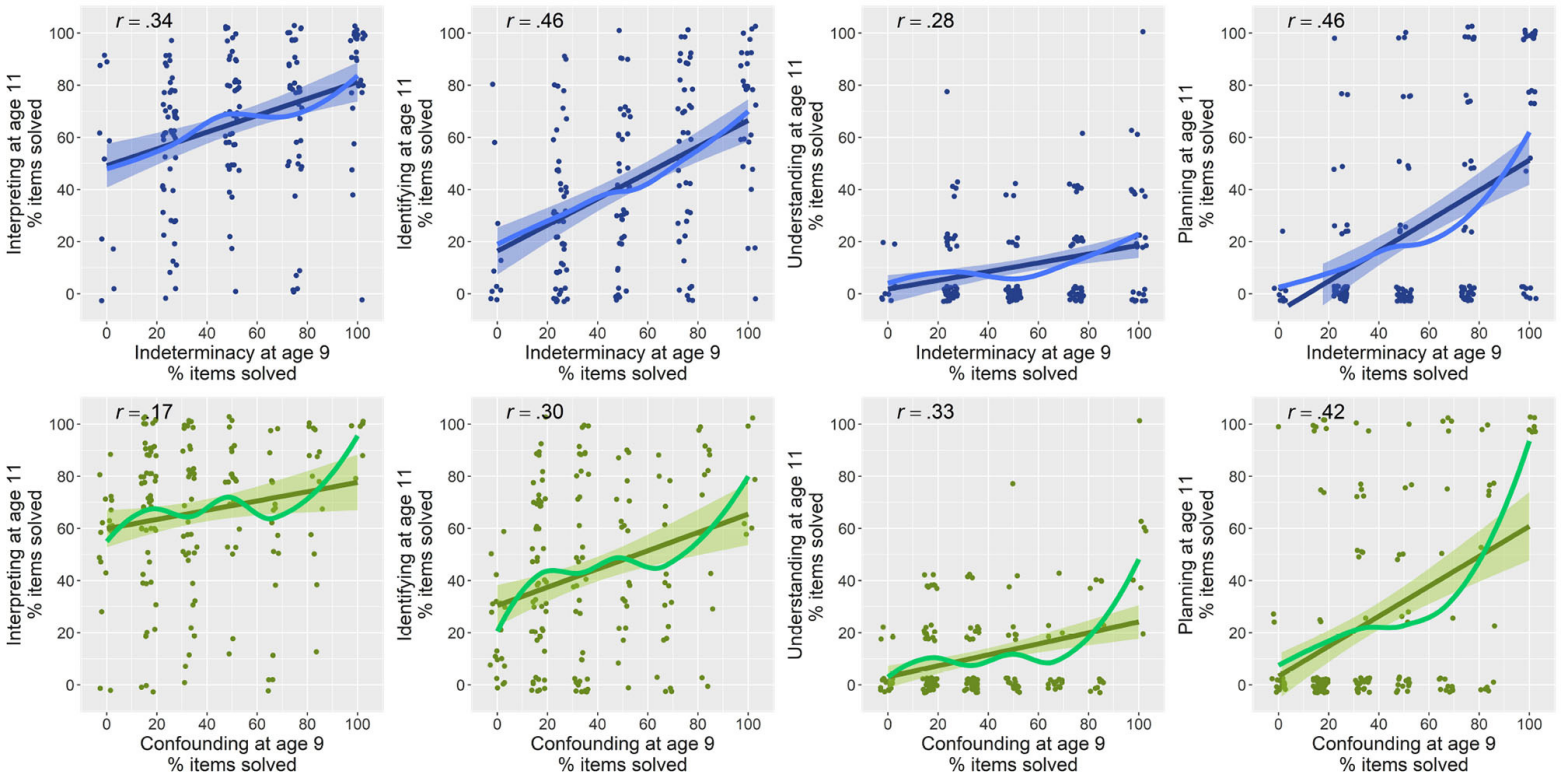
Relations between children's precursory skills and later CVS skills. First row shows relations of indeterminacy with later skills, and second row for confounding. Pearson correlations between variables are indicated in upper left of plots.

Associations between the precursor skills and the four later CVS sub-skills are depicted in Figure 7. All plots show the percentage of items solved for all four sub-skills of CVS. Lines with 95% confidence band indicate a linear trend, and lines without confidence band indicate the estimated nonlinear relation. The upper row shows the association between indeterminacy and the four sub-skills. The lower row shows the associations between confounding and the four sub-skills. According to these scatter plots, there are again some nonlinear relations between both precursor skills and the sub-skills of CVS. Hence, we fitted 16 additive mixed models. For each sub-skill, we estimated four models in line with the models described above. The results of the fitted models are summarized in Table 4. The eight models estimating both precursor skills as individual predictors (Models 1 and 2 for each sub-skill) indicated that indeterminacy alone could explain between 9.3 and 22.3% of children's later variation in individual CVS sub-skills (all *F*'s > 6.9, all *p*'s < 0.001). The most deviance could be explained for the sub-skill *planning*. Confounding could explain between 5 and 24.5%, whereas the most explained deviance was estimated regarding the sub-skill *understanding* (*F* = 14.91, *p* < 0.001). This result was significant. The lowest explained deviance was estimated for *interpreting* (*F* = 3.1, *p* = 0.28). This result was not significant. Taking into account the interaction between the two predictors, we found for all four models (each Model no. 3 in Table 4) significant main effects for the precursor skill indeterminacy (all *p*'s < 0.03). We found small significant interactions on the sub-skills *identifying, understanding, and planning* [identifying (*F* = 0.30, *p* = 0.02), understanding (*F* = 1.95, *p* < 0.001), planning (*F* = 0.7, *p* = 0.006)]. We did not find a significant interaction of the two predictors on interpreting (*F* = 0.14, *p* = 0.1).

**Table 4:** Relative model fit and explained deviance for the 16 fitted general additive mixed models.

**Model no**.	**Model description**	**AIC**	**BIC**	**% deviance explained**
Model 1 int	Indeterminacy	1,601	1,611	10.8
Model 2 int	Confounding	1,615	1631	05.0
Model 3 int	Indeterminacy × confounding	1,604	1,623	11.6
Model 4 int	Indeterminacy × confounding, covariates, teacher	1,592	1,668	27.3
Model 1 ide	Indeterminacy	1,628	1,637	20.6
Model 2 ide	Confounding	1,650	1,667	10.9
Model 3 ide	Indeterminacy × confounding	1,626	1,644	23.1
Model 4 ide	Indeterminacy × confounding, covariates, teacher	1,589	1,677	46.4
Model 1 und	Indeterminacy	1,449	1,463	09.3
Model 2 und	Confounding	1,419	1,437	24.5
Model 3 und	Indeterminacy × confounding	1,420	1,445	24.9
Model 4 und	Indeterminacy × confounding, covariates, teacher	1,396	1481	42.5
Model 1 pla	Indeterminacy	1,678	1,693	22.3
Model 2 pla	Confounding	1,680	1,697	21.6
Model 3 pla	Indeterminacy × confounding	1,662	1,688	30.9
Model 4 pla	Indeterminacy × confounding, covariates, teacher	1,658	1,732	39.0

*Covariates were reasoning ability and reading comprehension, including their interactions with indeterminacy and confounding on all four sub-skills of CVS as dependent variables (int, interpreting; ide, identifying; und, understanding; pla, planning). teacher indicates a random intercept across teachers*.

Finally, we also tested the robustness of both precursor skills regarding the covariates, cognitive abilities, and reading comprehension. We fitted four models, taking into account the main effects of both covariates and additionally a random intercept across teachers in order to control for the multilevel structure. Results show no interactions between the two predictors, but robust main effects for indeterminacy regarding all four sub-skills (all *p*'s < 0.02), indicating predictive value beyond shared variance with the covariates and the random intercept across teachers.

In sum, these results support the findings regarding mastery of overall CVS. Both precursor skill are substantial predictors for all four sub-skills of CVS. Indeterminacy shows stronger predictive value when taking into account children's covariates and classroom dependencies, and stronger predictive value for the sub-skill *interpreting*. Regarding the three other sub-skills, small interactions indicate that both precursor skills have predictive value beyond each other.

## 4. Discussion

We examined the predictive value of the precursor skills of understanding indeterminacy and confounding for CVS skills 2 years later. Our analyses show that both indeterminacy and confounding are good and robust predictors of later CVS mastery overall, yet the predictive value of indeterminacy seems to be more pronounced. Particularly regarding the four individual sub-skills of CVS, indeterminacy stands out as a strong predictor even when controlling for systematic classroom differences and covariates (cognitive abilities and reading comprehension). In addition, we found differences in the two precursor skills' predictive strength for the four CVS sub-skills. Although indeterminacy seems to be especially predictive of children's later skills in identifying and interpreting controlled experiments, the understanding of confounding appears to be slightly more relevant for later skills in planning controlled experiments, and in detecting the inconclusiveness of confounded comparisons (representing the sub-skill *understanding*). These results might be slightly unexpected, since we assessed *confounding* as precursor by a task (see Figure 1B) based on a so-called choice-task (Bullock et al., 2009). Solving a choice-task requires an ability which in our conceptualization receives the label *identifying* in fully developed CVS. A possible explanation for this finding might be that mastery of the tasks assessing later CVS skills requires skills going beyond *identifying*. The understanding of confounding shows stronger positive relations to all four sub-skills in the upper 20% of solved items (i.e., the associations are stronger for higher-scoring students). Hence, it shows stronger nonlinear relations than the understanding of indeterminacy. Finally, we found that the interactive predictive strength of the two precursor skills remains when taking into account children's more general cognitive development.

The pronounced yet not perfect predictive value of the two precursor skills indicates that although there is systematic stability in children's development of experimentation skills, new individual differences arise during age 9–11. In addition, the predictive value of precursor skills differed between the four CVS sub-skills. One reason for the varying findings across CVS sub-skills could arise from different requirements that the two precursor skills and the four CVS sub-skills pose on children. Solving an item representing the understanding of indeterminacy requires connecting the outcomes with the initial variables and rethinking the experiment in a deductive manner. Solving items representing the understanding of confounding demands an inductive way of thinking. In the latter, children had to choose different variable levels from a given sample in order to solve the item and thus are able to gain new insights of the experiment. Additionally, the comprehension of confounding requires understanding a central mechanism of the CVS by keeping all additional variables beyond the focal variable constant.

There were several nonlinear relations between the variables in our study. An intuitive explanation for this finding is that variation in lower scores is mostly caused by guessing, whereas variation in higher scores is where mastery of the precursor skills and the later CVS sub-skills really comes into play. Consequently, the nonlinear nature of various relations in this study might be attributed to a methodological issue caused by multiple choice-tasks. However, this does not mean that modeling the nonlinearity in the relations is superfluous. To the contrary, when such nonlinear effects are neglected in statistical models, this will lead to underestimation of the real predictive value of one skill for another. Consequently, we suggest researchers who use multiple choice-tasks with limited range in their study to examine and consider modeling nonlinear relations as well.

Solving items that comprise the sub-skill *understanding* demands understanding the indeterminacy of confounded experiments. *Planning* requires the full understanding of CVS: Without applying the CVS correctly, no conclusive experiments can be planned consistently. These two skills seem to be closer in their mechanisms to the understanding of confounding. Peteranderl (2019) found that solving planning items showed a bimodal distribution. Either the children failed or they succeeded in most of the cases. This could explain the strong positive relation in the upper 20% of solving confounding items regarding the planning sub-skill. This explanation is related to findings by Schwichow et al. (2016a) and Schwichow et al. (2020). Those researchers found that the sub-skill *understanding* appears to be much more difficult than the other sub-skills even for secondary school students. They concluded that *understanding* might premise mastering the other sub-skills. A stronger predictive value of the precursor skill of confounding for detecting the inconclusiveness of confounded experiments and the positive relation for the upper 20% of all sub-skills support the conclusion of Schwichow et al. (2020) that full development in all other sub-skills yields further development in more difficult sub-skills, in particular *understanding*.

By contrast, the sub-skill *interpreting* demands the ability to interpret the result of a conclusive comparison correctly, whereas the sub-skill *identifying* demands the ability to select a conclusive comparison according to an initial hypothesis. Both sub-skills have been assessed within the same item contexts. First, children had to select a comparison that was conclusive, and second, they had to interpret this comparison. In our understanding of CVS, it is not feasible to separate these two sub-skills conceptually, as it was done in tasks such as the choice-task by Bullock et al. (2009), Osterhaus et al. (2020), and Piekny and Maehler (2013). According to Peteranderl (2019), it is important to have an overarching understanding of both sub-skills, and the ability to combine them into the same task is decisive for correct understanding of CVS. Nevertheless, there were many children who solved the first task (identifying) but failed in the second (interpreting) and vice versa. This finding indicates that the ability to apply one sub-skill of CVS does not necessarily cause correct application of the other sub-skill. This could explain the differing associations of the sub-skills interpreting and identifying with the two precursor skills and the stronger predictive value of the precursor of indeterminacy on both sub-skills.

The findings of our study add to the multidimensional perspective regarding the dimensionality of scientific reasoning (Mayer et al., 2014; Koerber et al., 2015; Edelsbrunner and Dablander, 2018). Our findings endorse the perspective of both CVS and its precursors, which just make up a part of scientific reasoning, as multidimensional constructs (Chen and Klahr, 1999; Schwichow et al., 2016a). If CVS or its precursor skills are assessed or modeled as unidimensional constructs, important differences in the predictive value of different skills and their predictive interplay would get lost (see also Edelsbrunner et al., in Press). In addition, some of the intercorrelations between the different CVS sub-skills were only moderate, even on the latent level, as indicated by a confirmatory factor analysis. Moderate intercorrelations even when controlling for measurement error variance via latent variable modeling indicate that in an overall score, a substantial amount of differential information would get lost. We therefore suggest that when overall scores of CVS or even broader scientific reasoning are used for analyses, researchers should check whether thereby relevant information that would be visible in more detailed sub-scores is lost. In addition, theoretical models of scientific reasoning should acknowledge that conceptualizing scientific reasoning as being strongly dominated by an encompassing core of understanding of the theory-evidence relation might not be a fruitful direction for research.

In line with the results of Schwichow et al. (2016a, 2020), we found that the sub-skill *understanding* shows the lowest performance of all four sub-skills, reflecting that these items are also the most challenging ones for younger children. We also had multiple children in our sample that could not solve *identifying*-items but were rather successful on *planning*-items, with the two latent variables behind these tasks correlating 0.56. Apparently, also these two sub-skills do not perfectly inform about each other, although they seem theoretically related. We believe that based on these results, researchers should critically consider under which circumstances, and for which research questions, assessing or modeling CVS, its precursor skills, or broader scientific reasoning as unitary constructs might be useful, or rather lead to a loss of useful information. We do not argue against the usage of overall scores or unidimensional models in general. If, for example, researchers aim at modeling scientific reasoning as a competence that is equally represented by its constituents, an overall score or Rasch modeling might provide perfectly reasonable and informative approaches for achieving this aim. Our findings combined with prior studies (Edelsbrunner and Dablander, 2018; van Bork et al., 2019; VanderWeele and Batty, 2020) indicate that empirically, related questions might not be fully answered based on cross-sectional data, and that researchers should rather consider what models might be informative for the question at hand, pointing toward longitudinal examinations (or intervention studies) as a fruitful opportunity for examining dimensionality from a developmental perspective. A major reason is that longitudinal data provide the key for distinguishing between two reasons for an apparently (partially or more fully) unidimensional statistical structure: First, there can be a conceptual common core of scientific reasoning. Second, unidimensional structure can just appear because different skills influence each other over time; hence, unidimensionality arises that merely points toward developmental interplay (Van Der Maas et al., 2006).

Previous studies showed moderate to strong relations of facets of more general cognitive development with scientific reasoning (Morris et al., 2012; Piekny and Maehler, 2013; Mayer et al., 2014; van der Graaf et al., 2015, 2019; Osterhaus et al., 2017; Koerber and Osterhaus, 2019; Sande et al., 2019). Our results shows that the predictive value of both examined precursors on later mastery of CVS remains rather robust when taking into account general cognitive abilities and reading comprehension. Thus, new individual differences that arise when precursor skills develop into fully developed CVS cannot be explained by overarching more general cognitive development. Consequently, the development of CVS cannot be reduced to more general cognitive development, but also comprises domain-specific dynamics yielding CVS as an ability that is applicable in a domain-general manner similar to general reasoning, yet represents a distinct ability.

As a limitation of our study, it might be argued that posing the full CVS instrument from the second assessment on children twice, before and after the crucial developmental period, would have allowed more robust and additional insights. We agree with this point from a substantial perspective. However, posing a rather advanced instrument on children at age 9 might pose validity problems. The instrument might not work adequately in younger children, particularly with the *planning*-items that pose a rather complex task on children. We believe that it would be highly timely to develop an instrument that can be employed across a rather large period of childhood and beyond. In addition, we believe that validity issues, such as a lack of measurement invariance, might also arise because age 9–11 might not only be a period of increased quantitative development. Rather, this period might represent a time during which children more thoroughly restructure their knowledge base and skills regarding experimentation, representing qualitative development that would cause measurement issues when trying to assess the same skills over time.

Overall, our results shed more detailed light on previous findings (Sodian et al., 1991; Chen and Klahr, 1999; Bullock et al., 2009; Schwichow et al., 2016b) according to which children's understanding of CVS develops rapidly between age 9 and 11. Our longitudinal data suggest moderate individual stability in developing scientific reasoning skills. Earlier understanding of indeterminacy and confounding has clear yet limited predictive value for later mastery of CVS, going beyond that of more general cognitive development.

## Data Availability Statement

The datasets generated for this study are available on request to the corresponding author.

## Ethics Statement

The studies involving human participants were reviewed and approved by ETH Zurich. Written informed consent to participate in this study was provided by the participants' legal guardian/next of kin.

## Author Contributions

All authors listed have made a substantial, direct and intellectual contribution to the work, and approved it for publication.

## Conflict of Interest

The authors declare that the research was conducted in the absence of any commercial or financial relationships that could be construed as a potential conflict of interest.
